# Bioassays and Methodologies for Insecticide Tests with Larvae of *Trogoderma granarium* (Everts), the Khapra Beetle

**DOI:** 10.3390/insects10050145

**Published:** 2019-05-22

**Authors:** Frank H. Arthur, Michael J. Domingue, Deanna S. Scheff, Scott W. Myers

**Affiliations:** 1 USDA, ARS, Center for Grain and Animal Health Research, 1515 College Avenue, Manhattan, KS 66502, USA; deanna.scheff@ars.usda.gov; 2 Department of Entomology, Kansas State University, Manhattan, KS 66506, USA; michael.j.domingue@usda.gov; 3 Otis Laboratory USDA, APHIS, CPHST, 1398 West Truck Road, Buzzards Bay, MA 02542, USA; scott.w.myers@usda.gov

**Keywords:** residual control, insecticides, testing procedures, stored products

## Abstract

New insecticide treatment options would be beneficial for control programs for *Trogoderma granarium* Everts, the khapra beetle, in the United States. Two insecticides were evaluated, the Polyzone^®^ formulation of deltamethrin and a formulation of the insect growth regulator methoprene combined with deltamethrin and the synergist piperonyl butoxide. In the test with Polyzone^®^ deltamethrin, concrete arenas were treated with a low and high rate, and held outside, inside a shed, or inside a lab. Compared to storage in the lab, residue degradation increased slightly in the shed, and then further outside, as evidenced by greater larval survival and adult emergence. Across all environmental treatments, the high rate was more effective than the lower rate. For the combination methoprene product, the effect of food contact with treated surfaces was examined. When treating arenas with food and transferring the food to clean dishes, there was no immediate effect on larval survival, but there was a reduction in survival and emergence to the adult stage after one month. For both tests, larvae apparently often went into diapause after they were introduced onto the treatment arenas. Both treatments could be utilized in management programs if *T. granarium* infestations are detected.

## 1. Introduction

*Trogoderma granarium* (Everts), the khapra beetle, is one of the most destructive stored product pests of stored commodities and processed goods throughout the world [1]. It can infest a wide variety of products [1,2] and larvae can enter diapause and remain in that state for years when food resources are scarce [3]. The larva is the most destructive life stage, as the adults only live for 2–3 weeks with no or limited feeding and do not fly [4]. Most developed countries have quarantine regulations in place which require remedial action when this pest is detected in imported goods, but there are concerns regarding potential spread and introduction of *T. granarium* with increasing worldwide trade and commerce [5,6].

In the United States (US), the USDA Animal Plant Health and Inspection Service (APHIS) has the primary regulatory responsibility when quarantined insect species including *T. granarium* are detected [7]. The majority of khapra beetle detections in the US occur in passager baggage; however, it is also occasionally found in commercial shipments in a variety of commodities. When this occurs, the commodities are typically re-exported, but other options to mitigate risk of khapra beetle introduction include destruction or fumigation with methyl bromide. When an infestation of *T. granarium* is detected outside of a port environment, contact insecticides are frequently relied on for remedial actions [7]. The USDA-APHIS Center for Plant Health Science and Technology (CPHST) Otis Laboratory (Buzzards Bay, MA, USA) has been cooperating with scientists at the USDA-ARS Center for Grain and Animal Health Research (CGAHR) in Manhattan, KS, USA, to evaluate newer reduced-risk contact insecticides and insect growth regulators (IGRs) for control of *T. granarium*. Tests have been done by treating concrete surfaces with the label rates of the contact pyrethroid insecticides deltamethrin and cyfluthrin [8] or the IGRs methoprene and pyriproxyfen [9], and assessing residual efficacy from 0–3 months post-treatment.

Results generally show that pyrethroids will give control of adults, but larvae are harder to kill compared to adults and older larvae are more tolerant than younger larvae [10,11]. Exposure of larvae on surfaces treated with IGRs will limit eventual adult emergence of those exposed larvae. However, with both pyrethroids and IGRs, larvae must be provided with a food source during experiments to prevent death by starvation or inducing diapause in *T. granarium*. Also, continual exposure of those larvae, as opposed to short exposures and then removal to untreated arenas, is necessary for complete inhibition of adult emergence [8,12]. One other notable result from previous studies is that susceptibility of *T. granarium* to pyrethroids and IGRs may be similar in susceptibility to the related dermestids *Trogoderma variabile* (Ballion), the warehouse beetle, and *T. inclusum* (LeConte), the larger cabinet beetle [8,11]. The latter two species are both established in the US, so evaluating treatment efficacy is possible without the constraints of working indoors in an insect containment facility.

Most of the studies cited above employed standard laboratory techniques and methodologies for exposure of *T. granarium* larvae. There is a need to evaluate alternative techniques for assessing residual efficacy. A recent study by Arthur [13] described an experiment in which deltamethrin was applied to concrete arenas which were then held in an outside grain bin, a building, and a laboratory chamber. Efficacy was assessed by rapidity of knockdown of exposed adult *Tribolium castaneum* (Herbst), the red flour beetle. During the summer, residual efficacy decreased much faster on the arenas held inside the grain bin, suggesting that extreme temperatures led to faster degradation, and thus slower knockdown times and reduced efficacy. In a separate study, the impact of residual food material on efficacy of the pyrethroid cyfluthrin was also assessed using adult *T. castaneum* [12]. In that study, food that was put on a treated surface apparently absorbed the insecticide residues so that when the food was transferred to an untreated surface, exposed adult beetle were killed through contact with or ingestion of the residues absorbed from the food.

These same or similar techniques for assessing insecticidal efficacy against larval *T. granarium* may give a better indication of efficacy of new reduced-risk insecticides that more accurately reflects real-world conditions. Two separate but related studies were conducted in this experiment. In the first study, the objective was to evaluate residual efficacy of a new formulation of deltamethrin (Polyzone^®^), by treating concrete arenas and holding them in three different locations before bioassays were conducted. This was done to evaluate how efficacy declines in different environmental situations so that treatment application intervals can be appropriately estimated. In the second study, the objective was to evaluate a new insecticide formulation that contained methoprene, deltamethrin, and the synergist piperonyl butoxide, by examining the impact of food material on residual efficacy.

## 2. Materials and Methods

### 2.1. General Description

Both tests were conducted in the insect containment facility at the Otis Laboratory in Buzzards Bay, MA, US, the only site in the US where *T. granarium* can be reared under quarantine conditions. The laboratory colony at Otis was established in 2011 using adults and larvae collected from a market in Pakistan.

The colony was cultured in individual 0.95 L glass jars containing about 400 g of ground dog food, rolled oats, and other food material [8]. Jars were held in an environmental chamber at 30 °C. Four 10 × 6 cm pieces of laboratory “Precision Wipes” (Kimberly Clark, Irving, TX, USA) were placed in each jar, and larvae crawled onto the paper. When larvae were collected for testing, the paper was removed from the jar and placed in a tray that was under a hood inside an enclosed room within the facility, to limit potential escape of larvae.

### 2.2. Experiment 1: Deltamethrin (Polyzone^®^) Evaluation

This test was conducted using the deltamethrin Polyzone^®^ formulation, obtained from Bayer Crop Science (Whippany NJ, US). It was 4.75% Active Ingredient, 50 mg AI/mL, and label specifications allowed for application at a range of 7.5–45 mL in 3.8 L of water to cover 94 m^2^. Due to the inherent research limitations of working in the quarantine facility, we chose to examine just two rates, 15 and 45 mL, termed low and high rates (DL and DH), respectively, along with an untreated control (UTC) as a third treatment.

Individual concrete exposure arenas were created by mixing a dry concrete patching material (Rockkite, Heartland Products, Cleveland, OH, USA) with water to create a liquid slurry, and pouring this into the bottom portion of a plastic Petri dish (100 mm top lid dimension, 20 mm side height) to a depth of about 0.5 cm. This procedure has been described in detail in previous publications [8,9,11]. The bottom portion of the Petri dish measures about 62 cm^2^. A total of 180 arenas were created for the experiment. After the concrete dried (which took about 7 to 10 days, the sides of the lids were coated with Fluon^®^ (Northern Products, Woonsocket, RI, USA) to limit escape of larvae from the surface.

The arenas were treated as follows. The low rate was formulated by mixing 1.5 mL of the formulation with 23.5 mL of water (equivalent to the volume rate from the label) in each of 5 separate 25 mL flasks for five separate replicates. The area of the Petri dish was 62 cm^2^, so the equivalent volume rate to apply to that area in accordance with the label rate was about 0.27 mL, rounded up to 0.3 mL. Spray treatments were done using a Badger 100 artists’ airbrush (Badger Company, Franklin Park, IL, USA) to dispense the liquid onto an individual concrete arena. Twelve arenas were treated for each replicate. For the replicates in the high rate, 0.3 mL of the parent formulation was mixed with water in each of the 5 separate 25 mL flasks, and treatments were done as described above. Thus, there were 60 total arenas treated with the low rate and 60 arenas treated with the high rate. A third set of 60 arenas was treated only with 0.3 mL of distilled water each, to serve as untreated controls.

After the treatment arenas were completed, they were divided into three sets, each set containing four groupings of five replicates of each of the three treatments (untreated controls, low rate of deltamethrin, and high rate of deltamethrin). The experimental design was a factorial with three insecticide rates, three environmental holding conditions, and four monthly bioassays. One set of arenas was placed outside of the east wall of the main laboratory building, where they would be exposed to the wind, rain, and direct sunlight. A second set was placed on a shelf inside a greenhouse. The third and final set was held in a laboratory room under ambient conditions. The next day, one of the four groupings of arenas were removed from each of the three locations, brought inside the quarantine facility, and ten 3–4-week old larvae of *T. granarium*, along with about 500 g of diet, was placed in each arena. The arenas were covered with lids to prevent larvae from escaping and placed inside an environmental chamber at 30 °C and 60% r.h. After five days, the arenas were removed from the environmental chamber and larvae were examined and classified as live (active and mobile), affected by the insecticide (immobile with limited movement), or dead (unresponsive when probed). The arenas were returned to the environmental chamber, and after 30 days removed again and the numbers of live larvae, pupae, and adults were recorded and tabulated. The arenas were then frozen for two weeks at about −18 °C and then discarded.

After 1, 2, and 3 months post-treatment, bioassay and assessment procedures were repeated for each set of arenas held in the three locations (outside, greenhouse, lab counter). Data for the 5-day larval mortality assessments and the 30-day adult assessments were analyzed using Mixed Model Procedures in the Statistical Analysis System (Version 9.2, SAS Institute, Cary, NC, USA), with treatment, arena holding location, and bioassay month as the main effects, and replicate as a random effect. Variables of analysis were live larvae at the 5-day assessments, live adults at the 30-day assessments, and live larvae and adults combined at the 30-day assessments. Means were separated using Tukey’s Honestly Significant Difference (HSD) as an option in the Mixed Procedure in SAS.

### 2.3. Experiment 2: Methoprene + Deltamethrin + Piperonyl Butoxide Evaluation

The new formulation of methoprene, which also contains deltamethrin and the synergist piperonyl butoxide, was obtained from Central Life Sciences (Schaumberg, IL, USA). It contains 2.85% Active Ingredient (AI) methoprene, 1.2% AI deltamethrin, and 33.3% AI piperonyl butoxide, or 12.0, 27.4, and 320 mg AI/mL. The proposed application rate of this product is 180 mL in 3.8 L to cover 94 m^2^. Therefore, to formulate at this proposed rate, 0.5 mL of product was mixed with water in a 10 mL flask. The same concrete arenas were constructed as described for the deltamethrin test, and the volumetric spray rate remained at 0.3 mL of formulated spray per arena. In this test, there were just two treatments, untreated controls sprayed with 0.3 mL of distilled water and the insecticide as described above. The artist’s airbrush system described for the deltamethrin test was also used in this experiment.

The life stage of *T. granarium* used for this study was 3–4-week-old larvae. Five separate flasks were formulated as described above for each of the five replicates in 10 mL flasks. Eight arenas were needed for each replicate. Four arenas were kept clean when treated and four arenas contained about 500 g of the rearing diet when they were treated. Thus, after the treatment process was completed, there was a total of 40 clean treated arenas and 40 arenas that were treated with food material in the arenas. A companion set of 20 untreated arenas was treated with 0.3 mL of distilled water, again using a different airbrush. A third set of 20 untreated arenas was put with the others, as will be explained below. All arenas were then placed on a laboratory counter in ambient conditions.

One day after treatment, five of the original clean treated arenas and five of the food-treated arenas, along with the companion set of untreated arenas, plus 10 of the third set of untreated arenas, were removed and taken to one of the labs in the Quarantine room. The food from the food-treated arenas was transferred to one of the untreated arenas from the third set. Thus, there were now three exposure arenas for each treated and untreated replicate: the clean treated arena, the arena that was previously treated with the food inside but now with the food removed, and the third arenas which was untreated but now contained the treated food.

Ten *T. granarium* larvae were put in each arena, and the arenas were placed inside the incubator described for the deltamethrin study. After 5 days, the arenas were removed, and the larvae classified as live, affected, or dead using the criteria described in Experiment 1. The larvae and arenas were returned to the incubator, and after 30 days removed and individual *T. granarium* was classified as adults or live pupae or live larvae. This process was repeated at 1, 2, and 3 months post-treatment. Data were analyzed as described above for Experiment 1, using the Mixed Procedure of SAS. Means were separated using Tukey’s HSD test as an option under Proc Mixed.

## 3. Results

### 3.1. Experiment 1: Deltamethrin (Polyzone^®^) Evaluation

All main effects treatment, location where arenas were held, and month, and all associated interactions were significant at *p* < 0.001 for survival of *T. granarium* larvae after five days exposure on the arenas (Table 1). Even holding the arenas outside for just one day was enough to cause degradation of the low rate of deltamethrin, and there was no difference between the untreated controls (Table 2). Although the environmental exposure period was only one day, a rainstorm did occur overnight, which might have washed away or diluted the residues.

At one and two months, survival on arenas treated with the low rate of deltamethrin Polyzone^®^ was generally greater for larvae on the arenas held outside compared to those held in the shed or in the laboratory, but at month 3 there was no difference with respect to location. After month one, survival of larvae exposed on the high rate of deltamethrin that were held outside ranged from 92 to 98%. In contrast, survival of larvae on arenas held inside the shed or inside the laboratory was much lower (0 to 20%).

All main effects and interactions were significant (*p* < 0.001) for adult emergence 30 days after larvae were exposed on the arenas (Table 3). When data were analyzed for differences between treatments and locations, there were two occasions where larval diapause biased the results; untreated controls at month 1 for arenas held in the shed and month 3 for arenas treated with the high rate of deltamethrin and held outside (Table 4). However, in general adult emergence followed similar patterns to the five-day survival counts in that after month 1, adult emergence from exposed larvae was generally greater on those arenas held outside compared to the shed or the laboratory. Adult emergence was generally greater on arenas treated with the low rate of deltamethrin compared to the high rate, and there were occasions when emergence at the low rate on arenas held in the lab was greater than emergence on untreated controls.

Given the fact that diapause was occurring sporadically in the exposed larvae regardless of treatment, the data were re-analyzed by combining emerged adults at 30 days with live larvae. The live larvae could be distinguished from dead larvae, as the dead larvae were usually withered and discolored. This provided a clearer picture of the treatment effects (Table 5). After month 1 there was no difference between the two deltamethrin rates on arenas held outside, again indicating degradation of the deltamethrin residues on the arenas. In general, there was less adult emergence and live larvae on arenas treated with the high rate of deltamethrin and held in the shed or the lab, compared to the low rate. The combined percentage for adults ranged from 82 to 100% and was usually greater than the combined percentage for the low rate of deltamethrin on those arenas held in the shed or the lab.

### 3.2. Experiment 2: Methoprene + Deltamethrin + Piperonyl Butoxide Evaluation

The general ANOVA for survival of larvae after five days of exposure was significant at *p* < 0.001 for main effects treatment (untreated controls versus treatment with methoprene + deltamethrin + piperonyl butoxide (PBO), food condition (exposure of larvae on clean treated arenas, exposure on food-treated arenas, or on treated food transferred to untreated arenas). However, month and the treatment × month interaction was not significant (Table 6). Data were then combined by month to analyze for differences between treatment and food condition. Survival of larvae was always greater on untreated controls compared to the treatment at each food condition, and survival was usually greater on the transferred food compared to the other two food conditions (Table 7). However, the fact that there was at least some mortality on the transferred food was indicative of transfer of the insecticide residues as well.

The general ANOVA for adult emergence 30 days after larval exposure was significant at *p* < 0.001 for all main effects and the treatment by month and the treatment by food condition by month interactions (Table 8). For the analyses of treatment and food condition effects (Table 9), adult emergence was greater in the untreated controls compared to every comparison for food effects, and except for month 2, there was no difference in adult emergence between any of the three food conditions. Results for clean treated arenas with new food and food treated arenas with replaced food are discrepant with other months, but we have no explanation for that discrepancy other than perhaps the larvae used at this bioassay were slightly older than those used for other months. Data were also analyzed by combining adult emergence with live larvae after 30 days (Table 10), which again indicated some diapause induction in larvae but did not change the significance levels between treatments or food conditions; however; these data also indicate transfer of the insecticidal residues from the treated food to the untreated arenas (food transferred).

## 4. Discussion

According the product label, the deltamethrin Polyzone^®^ formulation creates a microscopic polymer film that binds to the surface and protects the active ingredient from weather, precipitation, irrigation, and mechanical abrasion. It is different from the Suspend^®^ and Centynal^®^ formulations of deltamethrin that do not have this technology, but the amount of active ingredient and label specifications for application as a residual surface treatment are the same for all three formulations. Deltamethrin Polyzone^®^ has been tested in a number of recent studies as a simulated barrier treatment for mosquitoes in outdoor situations and has been proven effective with increased residual persistence compared to standard commercial formulations of deltamethrin [14,15,16]. In our test with a stored product insect, which is the first test reported in the US with this formulation of deltamethrin, residual persistence was far less in our arenas held outdoors compared to those held in the shed or inside the lab. In a previous study, the Centynal^®^ formulation of deltamethrin was applied at equivalent label rates to concrete arenas held on the floor of a grain bin, inside a shed, or on a lab counter [13]. During tests conducted during the summer months, degradation occurred much faster on arenas held inside the grain bin compared to the two other sides, as evidenced by the increased time required for equivalent time to knock down and incapacitate adult *T. castaneum*. In that test, the reduced efficacy during the summer was attributed to greater high temperatures during the daytime hours in the grain bin compared to the other two locations. There was little sunlight inside the grain bin. This test is the first test reported in the scientific literature involving a stored product insect. In the current deltamethrin Polyzone^®^ test, the arenas held outside were subject to higher temperatures, but also to sunlight and effects of weathering and potential dilution due to rainfall. Also, controls for *T. granarium* are more likely to be implemented indoors rather than outdoors unless the surface was protected from environmental elements, so results from the shed and lab components of the current test show that deltamethrin Polyzone^®^ could potentially be used in control programs for *T. granarium*.

Another factor that could affect residual persistence is that concrete is porous, and many historical studies document low persistence of contact insecticides compared to metal, tile, or other non-porous surfaces [17,18]. Thus, to more accurately assess efficacy of the deltamethrin Polyzone^®^ formulation for outdoor use to control stored product insects, additional tests should be conducted with other insect species and life stages on different treated surfaces. Hence, caution is warranted with interpreting the results of the deltamethrin Polyzone^®^ on the arenas that were held outside, because many other residual insecticides will have low persistence on concrete surfaces compared to non-porous surfaces.

The study with the new combination methoprene formulation was designed to obtain information on translocation or absorption of the residues from the treated concrete surface. In the study involving cyfluthrin and food source on a treated surface, the arenas were treated first, then food was added to the arena, and it was clear that the food material absorbed residues from the surface [13]. When the food was transferred there was apparent toxicity towards adult *T. castaneum*, plus a decline in efficacy on the surface from which the food had been removed compared to a clean treated surface. In addition, in other studies with cyfluthrin or the insecticidal pyrolle chlorfenapyr in which adult *T. castaneum* or *Tribolium. confusum* Jaquelin du Val, the confused flour beetle, were used as the test insects, the presence of food material, either while beetles were exposed or after they were moved to an untreated surface, allowed for recovery from knockdown and reduced efficacy [19,20].

When studies are done with larvae, food must be provided so that the larvae in untreated controls will complete development to the adult stage, and thus provide a means for comparison with the insecticide treatments. In the current test, the food was treated in the arena so that it would be a barrier to insecticide deposition on the surface. In addition, in studies with the combination methoprene and larvae of *T. castaneum* and larvae of *T. confusum*, residues in food and extraneous material could be translocated to untreated areas [21,22], thereby removing the residues from the targeted treated area. In the tests cited above with adult flour beetles, sanitation was emphasized in conjunction with insecticide treatments applied for residual surface control, but the same emphasis on sanitation considered when and if IGRs are applied for control, since the IGRs are providing the residual efficacy.

One of the difficulties in conducting insecticide assessments on larvae of *T. granarium*, and the reason for examining the studies with deltamethrin and the combination methoprene together even though different methodologies were used, is the tendency for larvae to enter diapause [23]. There are many possible explanations for diapause induction in *T. granarium* and other dermestid larvae, including temperature, density, either low or high, or depletion of food resources [24]. In our tests, the ten larvae were introduced into the individual arenas and seemingly had enough food resources to complete development from larvae to adult, but diapause still occurred in untreated controls and the deltamethrin and combination methoprene treatments. Combining the emerged adults with live larvae at the 30-day counts was necessary in some cases to accurately assess treatment effects. Extending the assessment period was not feasible because it was apparent that the emerged adults in the untreated controls, and in some cases in the deltamethrin treatment involving the arenas held outside, had already mated because small larvae were present in the food material. In addition, 3-4-week old larvae were used for this test because of the sheer numbers needed for the individual bioassays. A more precise determination of the age or size range for bioassays may be necessary to obtain more conclusive results. Increasing replications for testing may also reduce variations caused by diapause. Some diapause would likely still occur even with a precise age/size determination. The factors causing diapause are in large part likely due to the conditions in the colony jars experienced before transfer, and thus difficult to control for without extensive consideration of such factors.

*Trogoderma granarium* was identified as one of the most serious pests of agriculture throughout the world [25], due to its wide host range and ability to diapause in the larval stage for extended time periods [26,27]. Larvae of *T. granarium* can be relatively tolerant to insecticides that are commonly used to control stored product beetles [28,29], thus newer reduced-risk insecticides may become more important in the near future. The results of this study show that both the deltamethrin Polyzone^®^ and the combination methoprene formulation will give residual control of *T. granarium* larvae when applied on a concrete surface in an indoor situation. Both insecticides show potential use in control programs when and if *T. granarium* infestations are detected in US entry ports and warehouses.

## 5. Conclusions

Both deltamethrin Polyzone^®^ and the combined methoprene-deltamethrin-piperonyl butoxide formulations will limit adult emergence from exposed larvae. However, the tendency for larvae to enter diapause is a confounding factor when assessing the results of bioassays, and just using adult emergence as an assessment factor may underestimate treatment effects. Our tests showed that at the final assessment period it was possible to differentiate live larvae from dead larvae by exposure to either insecticide, thus mitigating the effects of diapause.

## Figures and Tables

**Table 1:** Overall ANOVA (Proc Mixed, Statistical Analysis System, Version 9.4), live larvae after five days, for main effects treatment (untreated controls and the two deltamethrin rates), location where arenas were held (outside, shed, or lab), and month post-treatment (0, 1, 2, or 3). All main effects and interactions significant at *p* < 0.001.

Factor	F	df
Treatment (Treat)	646.9	2,143
Location (Loc)	258.4	2,143
Month	56.3	3,143
Treat × Loc	69.2	4,143
Treat × Month	19.2	6,143
Loc × Month	6.9	6,143
Treat × Loc × Month	16.4	12,143

**Table 2:** Percentage of live *Trogoderma granarium* larvae (means ± SE) five days after being exposed on untreated arenas or arenas treated with low and high rates of deltamethrin (D) Polyzone^®^. Arenas were held either in the laboratory, in an outside area, or inside a shed. Bioassays were conducted 1 day after treatment (month 0) and at 1, 2, and 3 months post-treatment.

Bioassays	Arena Location	% Live *T. granarium* Larvae		
		Untreated	D-Polyzone^®^ Low	D-Polyzone^®^ High
Month 0	Lab	100 ± 0.0 aA	8 ± 4.9 bB	2 ± 2.0 aB
	Outside	100 ± 0.0 aA	84 ± 11.2 aA	6 ± 4.0 aB
	Shed	98 ± 2.0 aA	22 ± 9.7 bB	0 ± 0.0 aC
Month 1	Lab	100 ± 0.0 aA	28 ± 10.7 cB	16 ± 6.0 bB
	Outside	100 ± 0.0 aA	96 ± 2.5 aA	92 ± 3.7 aA
	Shed	100 ± 0.0 aA	44 ± 5.1 bB	10 ± 7.7 bC
Month 2	Lab	98 ± 2.0 aA	22 ± 7.3 cB	16 ± 6.0 bB
	Outside	100 ± 0.0 aA	98 ± 2.0 aA	98 ± 2.0 aA
	Shed	100 ± 0.0 aA	64 ± 11.2 bB	14 ± 7.4 bC
Month 3	Lab	100 ± 0.0 aA	84 ± 2.5 aA	0 ± 0.0 cB
	Outside	100 ± 0.0 aA	92 ± 5.8 aA	98 ± 2.0 aA
	Shed	100 ± 0.0 aA	84 ± 2.5 aB	20 ± 4.4 bC

**Table 3:** Overall ANOVA (Proc Mixed, Statistical Analysis System, Version 9.4), for percentage of live adults 30 days after larvae were placed on the arenas, and percentage of live adults plus live larvae, for main effects treatment (untreated controls and the two deltamethrin rates), location where arenas were held (outside, shed, or lab), and month post-treatment (0, 1, 2, or 3). All main effects and interactions significant at *p* < 0.001.

	Factor	F	df
% of Live Adults	Treatment (Treat)	214.3	2,143
	Location (Loc)	29.3	2,143
	Month	13.0	3,143
	Treat × Loc	5.2	4,143
	Treat × Month	7.3	6,143
	Loc × Month	19.0	6,143
	Treat × Loc × Month	4.5	12,143
% of live adults + live larvae	Treatment (Treat)	403.1	2,143
	Location (Loc)	69.4	2,143
	Month	24.1	3,143
	Treat × Loc	39.3	4,143
	Treat × Month	9.0	6,143
	Loc × Month	11.3	6,143
	Treat × Loc × Month	6.1	12,143

**Table 4:** Percentage of adult *T. granarium* (means ± SE) emerging from larvae exposed on untreated arenas or arenas treated with low and high rates of deltamethrin Polyzone^®^. Arenas were held either in the laboratory, in an outside area, or inside a shed. Bioassays were conducted 1 week after treatment and at 1, 2, and 3 months post-treatment ^1^.

Bioassays	Arena Location	% Adult *T. granarium* ^1^		
		Untreated	D-Polyzone^®^ Low	D-Polyzone^®^ High
Month 0	Lab	76 ± 15.0 aA	58 ± 8.6 aA	6 ± 4.0 aB
	Outside	76 ± 6.8 aA	44 ± 13.3 aA	6 ± 4.0 aB
	Shed	84 ± 4.0 aA	34 ± 10.3 aB	4 ± 4.0 aC
Month 1	Lab	86 ± 6.8 aA	42 ± 8.6 cB	2 ± 2.0 bC
	Outside	88 ± 9.6 aA	98 ± 2.2 aA	92 ± 3.7 aA
	Shed	50 ± 10.4 bA ^2^	68 ± 10.2 bA	6 ± 2.4 bB
Month 2	Lab	72 ± 13.2 bA	46 ± 9.3 bA	0 ± 0.0 bB
	Outside	100 ± 6.3 aA	90 ± 0.0 aA	82 ± 9.6 aA
	Shed	90 ± 5.4 aA	62 ± 13.9 bB	12 ± 8.0 bC
Month 3	Lab	80 ± 7.1 aA	92 ± 3.7 aA	22 ± 8.0 aB
	Outside	80 ± 3.2 aA	82 ± 4.9 aA	0 ± 0.0 bB ^3^
	Shed	94 ± 2.4 aA	88 ± 5.8 aA	20 ± 7.7 aB

^1^ Means for treatment by location and month (rows), followed by different capital letters, and means for location by treatment (columns), followed by different lower-case letters, are significant (*p* < 0.05, Tukey’s HSD test under Proc Mixed in SAS). ^2^ About half of the exposed larvae still in the larval stage but alive. ^3^ All exposed larvae still in the larval stage but alive.

**Table 5:** Percentage of live larvae plus adult *T. granarium* (means ± SE) emerging from larvae exposed on untreated arenas or arenas treated with low and high rates of deltamethrin Polyzone^®^, 30 days after introduction of larvae. Arenas were held either in the laboratory, in an outside area, or inside a shed. Bioassays were conducted 1 week after treatment and at 1, 2, and 3 months post-treatment.

Bioassays	Arena Location	% Of Live Larvae and Adult *T. granarium* ^1^		
		Untreated	D-Polyzone Low	D-Polyzone High
Month 0	Lab	98 ± 2.0 aA	72 ± 10.7 aB	10 ± 6.3 aB
	Outside	100 ± 0.0 aA	64 ± 13.3 aA	6 ± 4.0 aB
	Shed	96 ± 4.0 aA	48 ± 7.3 bB	4 ± 4.0 C
Month 1	Lab	98 ± 6.7 aA	58 ± 5.8 cB	2 ± 2.0 bB
	Outside	98 ± 2.0 aA	100 ± 0.0 aA	100 ± 0.0 aA
	Shed	100 ± 0.0	78 ± 5.8 bB	6 ± 2.4 bC
Month 2	Lab	94 ± 8.7 aA	66 ± 8.7 bB	0 ± 0.0 cC
	Outside	100 ± 0.0 aA	90 ± 0.0 aA	84 ± 9.3 aA
	Shed	92 ± 3.7 aA	76 ± 10.8 bB	12 ± 8.0 bC
Month 3	Lab	98 ± 2.0 aA	94 ± 4.0 aA	24 ± 8.1 cB
	Outside	82 ± 2.0 aA	96 ± 4.0 aA	92 ± 5.8 aC
	Shed	100 ± 0.0 aA	90 ± 6.3 aB	28 ± 9.2 bC

^1^ Means for treatment by location and month (rows), followed by different capital letters, and means for location by treatment (columns), followed by different lower-case letters, are significant (*p* < 0.05, Tukey’s HSD test under Proc Mixed in SAS).

**Table 6:** Overall ANOVA (Proc Mixed, Statistical Analysis System, Version 9.4), live larvae after 5 days, for main effects treatment (untreated controls versus the methoprene-deltamethrin-piperonyl butoxide formulation) condition (larvae exposed on the clean treated arenas with new food, the food-treated arenas with replaced food, and the untreated arenas with transferred treated food, and month post-treatment (0, 1, 2, or 3).

Factor	F	df	*p*
Treatment (Treat)	299.4	1,95	<0.001
Condition (Cond)	9.5	2,95	<0.001
Month	0.4	3,95	0.780
Treat × Cond	12.1	2,95	<0.001
Treat × Month	1.0	3,95	0.390
Cond × Month	3.3	6,95	0.006
Treat × Cond × Month	4.4	6,95	<0.001

**Table 7:** Percentage for live *T. granarium* larvae (means ± SE) five days after being exposed on untreated concrete arenas clean concreate arenas treated with the pyrethrin + methoprene + piperonyl butoxide formulation (IGR) arenas in which 500 g of diet was placed in the arenas prior to treatment, or arenas in which the food was removed and placed on untreated arenas. Bioassays conducted at one day after treatment (Month 0) or after 1, 2, and 3 months post-treatment. Month not significant in overall ANOVA (Table 6), data combined for month.

Arena Condition	% Live *T. granarium* Larvae ^1^	
	Untreated	IGR
Clean Treated	99.5 ± 0.0 aA	56.5 ± 3.9 bB
Food Treated	100.0 ± 0.5 aA	58.8 ± 3.9 bB
Food Transferred	98.5 ± 0.8 aA	77.5 ± 4.1 aB

^1^ Means for treatment by food condition (columns), followed by different lower-case letters, and means for treatment (rows), followed by different capital letters, are significant (*p* < 0.05, Tukey’s HSD test for food condition, and *t*-test for treatment, under Proc Mixed in SAS).

**Table 8:** Overall ANOVA (Proc Mixed, Statistical Analysis System, Version 9.4) for percentage of live *T. granarium* adults 30 days after larvae were placed on the arenas and the percentage of live adults + live larvae after 30 days. Main effects were treatment (untreated controls versus the pyrethrin + methoprene + piperonyl butoxide formulation (IGR), condition (larvae exposed on the clean treated arenas, the food-treated arenas, and the untreated arenas with transferred treated food), and month post-treatment (0, 1, 2, or 3).

	Factor	F	df	*p*
% of Live Adults	Treatment (Treat)	1899.4	1,95	<0.001
	Condition (Cond)	10.2	2,95	<0.001
	Month	17.1	3,95	<0.001
	Treat × Cond	3.0	2,95	0.054
	Treat × Month	8.5	3,95	<0.001
	Cond × Month	2.0	6,95	0.085
	Treat × Cond × Month	8.0	6,95	<0.001
% of Live Adults + Live Larvae	Treatment (Treat)	1182.4	1,95	<0.001
	Condition (Cond)	10.9	2,95	<0.001
	Month	3.4	3,95	0.020
	Treat × Cond	9.5	2,95	<0.001
	Treat × Month	2.1	3,95	0.107
	Cond × Month	1.8	6,95	0.108
	Treat × Cond × Month	4.1	6,95	<0.001

**Table 9:** Percentage of live adult *T. granarium* (means ± SE) emerging from the larvae exposed on the clean treated arenas with new food, the food-treated arenas with replaced food, and the untreated arenas with transferred treated food, as described in Table 6. Insecticide (IGR) as previously described. Bioassays conducted at one day after treatment (Month 0), or after 1, 2, and 3 months post-treatment. Adult emergence assessed 30 days after larvae were introduced onto the treated and untreated arenas.

Bioassays	Arena Condition	% Of Live Adult *T. granarium* ^1^	
		Untreated	IGR
Month 0	Clean Treated	98 ± 2.0 aA	0 ± 0.0 aB
	Food Treated	82 ± 9.2 aA	0 ± 0.0 aB
	Food Transferred	82 ± 4.9 aA	0 ± 0.0 aB
Month 1	Clean Treated	90 ± 4.4 aA	10 ± 7.7 aB
	Food Treated	100 ± 0.0 aA	0 ± 0.0 aB
	Food Transferred	84 ± 4.0 bA	0 ± 0.0 aB
Month 2	Clean Treated	98 ± 2.0 aA	36 ± 5.1 aB
	Food Treated	88 ± 5.8 aA	50 ± 8.4 aB
	Food Transferred	98 ± 2.0 aA	0 ± 0.0 bB
Month 3	Clean Treated	96 ± 4.0 aA	16 ± 11.2 aB
	Food Treated	100 ± 0.0 aA	6 ± 4.0 aB
	Food Transferred	96 ± 2.4 aA	4 ± 2.4 aB

^1^ For each month, means for percentage adult emergence from larvae exposed by food condition (columns), followed by different lower-case letters, and means for treatment (rows), followed by different capital letters, are significant (*p* < 0.05, Tukey’s HSD test for food condition, and *t*-test for treatment, under Proc Mixed in SAS).

**Table 10:** Percentage of live adult *T. granarium* (means ± SE) emerging from the exposed larvae, plus live larvae, on the clean treated arenas with new food, the food-treated arenas with replaced food, and the untreated arenas with transferred treated food. Insecticide treatment as previously described (IGR). Bioassays conducted at one day after treatment (Month 0), or after 1, 2, and 3 months post-treatment.

Bioassays	Arenas	% Of Live adult and Larval *T. granarium* ^1^	
		Untreated	IGR
Month 0	Clean Treated	100 ± 0.0 aA	10 ± 5.40 bB
	Food Treated	92 ± 8.0 aA	32 ± 11.1 aB
	Food Transferred	96 ± 2.4 aA	8 ± 5.8 aB
Month 1	Clean Treated	98 ± 2.2 aA	16 ± 9.2 b
	Food Treated	100 ± 0.0 aA	14 ± 6.0 bB
	Food Transferred	92 ± 2.2 bA	17 ± 5.5 bB
Month 2	Clean Treated	100 ± 0.0 aA	40 ± 5.5 aB
	Food Treated	96 ± 4.0 aA	54 ± 8.7 aB
	Food Transferred	100 ± 2.0 aA	0 ± 0.0 bB
Month 3	Clean Treated	100 ± 0.0 aA	24 ± 9.8 aB
	Food Treated	100 ± 0.0 aA	28 ± 8.6 aB
	Food Transferred	98 ± 2.0 aA	6 ± 2.4 aB

^1^ For each month, means for percentage adult emergence from exposed larvae, plus the percentage of live larvae, exposed by food condition (columns), followed by different lower-case letters, and means for treatment (rows), followed by different capital letters, are significant (*p* < 0.05, Tukey’s HSD test for food condition, and *t*-test for treatment, under Proc Mixed in SAS). IGR.
